# Review of Strategies to Promote Rumen Development in Calves

**DOI:** 10.3390/ani9080490

**Published:** 2019-07-26

**Authors:** Qiyu Diao, Rong Zhang, Tong Fu

**Affiliations:** 1 Key Laboratory of Feed Biotechnology of the Ministry of Agriculture, Feed Research Institute, Chinese Academy of Agricultural Sciences, Beijing 100081, China; 2 Precision Livestock and Nutrition Unit, Gembloux Agro-Bio Tech, University of Liège, Passage des Déportés, 2, 5030 Gembloux, Belgium; 3 College of Animal Science and Veterinary Medicine, Henan Agricultural University, Zhengzhou 450002, China

**Keywords:** calves, rumen, epithelium, microbiota, diet, feed additives, feeding management

## Abstract

**Simple Summary:**

The rumen is an important digestive organ that plays a key role in the growth, production performance and health of ruminants. Promoting rumen development has always been a key target of calf nutrition. Current research reveals that an early feeding regime and nutrition have effects on rumen development and the establishment of rumen microbiota. The effects may persist for a long time, and consequently, impact the lifetime productive performance and health of adult ruminants. The most sensitive window for rumen manipulation may exist in the postnatal and weaning period. Thus, the early feeding regime and nutrition of calves deserve further research. The establishment of the rumen bacterial community is a mysterious and complex process. The development of microbial 16S rDNA gene sequencing and metagenome analysis enables us to learn more about the establishment of rumen microbes and their interactions in host gastrointestinal (GI) tract development.

**Abstract:**

Digestive tract development in calves presents a uniquely organized system. Specifically, as the rumen develops and becomes colonized by microorganisms, a calf physiologically transitions from a pseudo-monogastric animal to a functioning ruminant. Importantly, the development of rumen in calves can directly affect the intake of feed, nutrient digestibility and overall growth. Even minor changes in the early feeding regime and nutrition can drastically influence rumen development, resulting in long-term effects on growth, health, and milk yields in adult cattle. Rumen development in newborn calves is one of the most important and interesting areas of calf nutrition. This paper presents a comprehensive review of recent studies of the gastrointestinal (GI) tract development in calves. Moreover, we also describe the effect of the environment in shaping the GI tract, including diet, feed additives and feeding management, as well as discuss the strategies to promote the physiological and microbiological development of rumen.

## 1. Introduction

Rearing healthy calves is very important as it can have a significant impact on their growth and milk production performance in adult life. Adequate calf development is therefore crucially important for the entire dairy industry. Calves are challenged by a series of stress factors after they are born, including changes in their surroundings. Specifically, the living environment changes from the sterile uterus to natural outside conditions, in addition to changes in nutrition from that provided by the mother to the digestion and absorption of feed by calves themselves. However, due to the poor immunity and the incomplete development of the digestive system in young calves, any interference from the external environment or changes to the nutrition can drastically affect the development of calves [1]. Some of the problems include diarrhea and slow weight gain, as well as respiratory tract disease, which can lead to high levels of morbidity and mortality, and pose significant challenges to breeding. 

## 2. Rumen Development 

Compared with monogastric animals, the forestomaches of ruminants have a specialized structure and function, which results in differences in digestion and physiology between ruminants and monogastric animals. Moreover, calves have additional unique systems that are present in their digestive tract during their development. At birth, the rumen is not completely developed, and significant changes in rumen development have to occur first before the calves can digest dry feed to guarantee their own growth needs. The specific changes include the development of the rumen organ and rumen epithelium, and the establishment of rumen microbiota. Understanding rumen development in newborn calves is one of the most important focus areas of calf nutrition.

### 2.1. Rumen Organ Development

The digestive system of young ruminants begins to develop during the embryonic period. For example, the stomach chambers are visible by day 56 in bovine embryos [2]. At birth, the weights of reticulorumen, omasum, and abomasum account for 38%, 13%, and 49% of the entire stomach weight, respectively [3]. By eight weeks of age, these proportions change to 61.23%, 13.40%, and 25.37% of the stomach weight, respectively [1]. Finally, at 12–16 weeks of age, they reach 67%, 18%, and 15% of the stomach weight, respectively [1,3] (Table 1). The esophageal groove, namely the rumoreticuler groove, is one of the unique features inside the gastrointestinal (GI) tract of calves. The majority of the liquid feed, such as colostrum, whole milk and milk replacer (MR), can bypass the rumen, reticulum and omasum, and flow directly into the abomasum as a result of the reflex closure of the esophageal groove. The abomasum of newborn calves is the only fully developed and functional stomach, and is also the most important digestive organ for calves at birth. The digestion of fat, carbohydrates, and protein is predominantly dependent on the digestive enzymes secreted by the abomasum and small intestine, which is similar to the digestive system in monogastric animals. Over time, with the increase in dry feed intake, the rumen begins to develop and starts to play more important digestive roles.

### 2.2. Rumen Epithelium

The ruminal epithelium performs many important functions and plays the key role in rumen development, including absorption, transportation, short-chain fatty acid metabolism, and protection. The proliferation and growth of the rumen squamous epithelium promotes the growth of papillae length and width, and increases the thickness of the interior rumen wall [4]. Work by Lesmeister and coworkers (2004) [5] considered the papillae length of the rumen as the most important factor for the evaluation of rumen development, followed by the papillae width and rumen wall thickness. However, papillae per square centimeter is not used as an indicator of rumen development. 

Newborn calves have a smooth epithelium with no prominent papillae. Calves fed solely with liquid feed have been shown to have limited rumen development characterized by decrease in rumen weight, papillary growth, degree of keratinization, pigmentation and musculature development [6,7]. Of note, increased intake of solid feed contributes to the rapid development of ruminal fermentation. As calves consume more starter feed, rumen digesta pH decreases, whereas volatile fatty acid (VFA) concentration gradually increases during the first two months. The molar proportion of acetate decreases during the first two months, and then starts to increase until nine months of age as forage intake increases [1,8]. The presence and absorption of VFAs in the rumen provides chemical stimuli required for the proliferation of rumen epithelium [6,9]. Importantly, intraruminal administration of acetate, propionate, and butyrate can stimulate the growth of rumen epithelium in young ruminants, with the effect of butyrate being the most prominent, followed by propionate [4,6]. Studies suggest that rumen papilla proliferation is associated with increased blood flow through the rumen wall [10,11] and a direct effect of butyrate and propionate on gene expression [12]. 

Despite many studies indicating that VFA can promote the development of rumen epithelium in vivo, the in vitro results suggest the opposite. For example, butyrate treatment decreases DNA synthesis of rumen epithelial cells in culture [13], while the proliferation of rumen epithelial cells is inhibited by rumen fluid in vitro [14]. The divergent in vivo and in vitro response may be linked with an indirect hormonal response to VFA metabolites. Several hormones, such as insulin, pentagastrin, and glucagon, have been implicated as possible VFA mediators that stimulate rumen epithelial proliferation [12,15]. A previous study by Baldwin (1999) reported that proliferation rates of rumen epithelial cells induced by insulin, epidermal growth factor, and insulin-like growth factor (IGF-1) were 75%, 97% and 96%, respectively [16]. Importantly, other studies also suggested that insulin, epidermal growth factor, and IGF-1 can overcome the inhibitory effect of butyrate [16,17].

### 2.3. Ruminal Microbiota 

At birth, the GI tract of young ruminants is sterile. During the first hours of life, the forestomach becomes rapidly colonized with an abundant bacterial population. The neonates acquire bacteria from the dam, partners, feed, housing and environment. The early gut microbes of suckled lambs were mainly derived from the mother’s teats (43%) and ambient air (28%), whereas those of bottle-fed lambs were dominated by bacteria from the mother’s vagina (46%), ambient air (31%), and the sheep pen floor (12%) (Bi et al., 2019) [18]. Facultative anaerobes such as *Streptococcus* and *Enterococcus* are the early colonizers of rumen, which convert rumen to a fully anaerobic environment to promote the rapid establishment of strictly anaerobic bacteria [19]. By two days of age, the rumen microflora reaches 10^9^ cells/mL with strictly anaerobic bacteria being predominantly found in the rumen of lambs [20]. The aerobic and facultatively anaerobic bacteria were 10- to 100-fold lower than the strictly anaerobic bacterial count observed during the first week, which continued to decrease afterwards [20].

Compared to older animals, the abundance of phylum Bacteroidetes was significantly lower in one-day-old calves and was mainly composed of the genus *Bacteroides*, whereas older animals were mainly colonized with *Prevotella* [19]. Work by Malmuthuge and coworkers (2014) [21] reported that the rumen contents of three-week-old calves contained a similar level of *Bacteroides* (15.8%) and *Prevotella* (15.1%), which may suggest that starter feed can propel rumen microbiome development to more mature status. The presence of cellulolytic and methanogenic bacteria was observed in lambs at three–four days of age, and the population of these bacteria reached a level similar to that observed in mature sheep within seven days of age [20]. Study by Jami and colleagues (2013) [19] reported that cellulolytic bacteria and other bacterial species important to rumen function can be detected as early as one day after birth. Thus, the establishment of these rumen bacteria occurs long before young ruminants have access to concentrated feed or forage. Dill-McFarland and coworkers (2017) [22] indicated that calves sampled a few days after weaning had a more diverse rumen community compared to calves sampled during weaning. Several fungal operational taxonomic units (OTUs) observed in weaned calves are also present in adults. As fungi mainly colonize fibrous solids, this may suggest an introduction of forage allows previously low-abundant or transient fungi to persist and multiply.

The rumen bacterial population of two-week-old calves fed milk replacer (MR) was reported to contain 45 bacterial genera belonging to 15 phyla [23]. Similarly, 47 bacterial genera belonging to 13 phyla were observed in the three-week-old calves [21]. Interestingly, the rumen microbiota of the two-week-old calves has more heterogeneous microbiota and harbors more abundant yet transient bacterial species and genera compared to calves at 42 days of age [23]. Another study suggested that the diversity and intra-group similarity of rumen microbiota increases with age, suggesting a transition from a heterogeneous and less distinct community to a more homogeneous and diverse mature bacterial population [19]. This is further supported by a recent study, where gut communities showed higher alpha-diversity but lower beta-diversity with age [22]. Co-habitation facilitates individuals to acquire a shared microbiota [24]. The rumen microbiota was similar in weaned and adult goats that were co-housed pre-weaning [25]. This may also contribute to a convergence toward a similar microbiota in the adult animals.

The composition of the rumen bacterial community varied significantly among individual calves, suggesting a strong host-microbiota specificity in the rumen [19,23]. Similarly, the communities of archaea and fungi in rumen varied considerably among individuals [22,26]. This may suggest that the composition of the rumen microbial community is associated with the physiological condition of the host [19]. Moreover, work by Mayer and coworkers (2012) [27] found that fecal microbial composition was more similar between twin calves than between siblings, implying that host genetics partly define individual gut microbial composition. 

Additionally, the bacterial composition was different among the gastrointestinal tract regions and between mucosa- and digesta-associated communities [21]. Colonization of calf rumen starts early in life with a distinct segregation of bacteria between digesta and epithelial surfaces. Similarly, the methanogen community also varies along the gastrointestinal tract [26]. This indicates that previous studies on fecal samples cannot adequately represent the complexity of the gut microbiome. Future studies should focus on both mucosa- and digesta-associated communities in rumen directly.

## 3. Strategies to Promote Rumen Development

Strategies to promote morphological structure and metabolic function of rumen in pre-ruminants are an ongoing issue which greatly attracts a lot of attention from the scientific community. Numerous studies and approaches attempt to modulate rumen fermentation and the microbial community in young ruminants to accelerate rumen development. These approaches include alteration of diet composition and physical forms, addition of new types of feed additives, and introduction of variables in the feeding management.

### 3.1. Diet

#### 3.1.1. Liquid Feed

Liquid feed may affect plasma concentration of hormones and growth factors, such as insulin and IGF-1, which play important roles in stimulating proliferation of rumen epithelial cells [16,28]. Colostrum contains many biologically active substances, mainly polypeptide growth factors and steroid hormones, including insulin, IGF-1, and transforming growth factor (TGF). Intake of colostrum has been associated with the development, digestion, and absorption ability of the GI tract in the newborn calves [16,28]. Moreover, a whole milk calf diet was shown to have a positive effect on milk yield during the first lactation of the adults compared to calves fed an MR diet. These results highlight the importance of biologically active milk-borne factors [29].

Soybean protein can be used as an alternative to milk protein in formulating MR [30]. Previous studies suggested that MR formulated with soy proteins can negatively affect the development of the small intestine [31,32]. The abomasal pH declines more slowly and pH is higher in calves fed MR containing soy flour compared to calves given whole milk [33,34,35]. Decreasing the pH of MR emulsion by addition of an acidifier reduces the pH of digesta pH in the rumen, reticulum, and omasum. Specifically, pH reduction of MR emulsion was found to be beneficial for the development of ruminal epithelium [36]. Work by Górka and coworkers (2011a) [37] reported a shorter papillae length of the cranial dorsal sac in calves fed MR compared to calves fed whole milk, and noted positive relationships between reticulorumen weight and small intestine weight, or with brush border enzyme activities. There is a close relationship between the development of the rumen and the small intestine. Importantly, different types of liquid feed affect the development of the small intestine, the intake of solid feed later in life, as well as the growth and metabolic status of calves, thereby indirectly affecting the development of forestomaches [37]. Enhancing the nutrition level of MR in calves induces changes in the expression of genes coding for proteins directly influencing rumen epithelial growth [38]. Moreover, liquid feed may flow into the rumen due to the closure of the esophageal groove. This can occur even in calves that are not clinically defined as rumen drinkers. Specifically, in veal calves that received large amounts of milk, the amount of leakage liquid was approximately 14–35%, which may induce ruminal and metabolic acidosis in a clinical case [39,40].

#### 3.1.2. Starter Feed

Feeding readily fermentable carbohydrates to calves increases VFA production in the rumen, which is necessary to stimulate the development of rumen epithelium [41,42]. Calves fed milk-only diet during the first three weeks present with a different microbial community in their GI tract and feces compared to calves given milk and solid feed [43]. Diets differing in carbohydrate composition lead to differences in rumen fermentation patterns and VFA profiles which may have a variable effect on rumen development [44,45]. For example, high concentrations of ruminal ammonia, acetate, propionate, and butyrate were detected in calves fed corn- and wheat-based diets compared to calves fed barley- and oat-based diets. Moreover, the forestomach weight and papillae growth were greater in calves fed corn- and wheat-based diets [46]. The mucosal thickness was greater in veal calves fed starch- and pectin-based diets compared to calves on neutral detergent fiber (NDF)-based diets, however, a higher incidence of poorly developed mucosa was observed in calves fed starch-based diet than in animals fed pectin- and NDF-based diets [40]. It was reported that the stimulatory effects of VFAs are different, with butyrate being most stimulatory followed by propionate and then acetate [4,6]. Butyrate provides energy required for rumen wall thickening, formation of papillae and stimulating capillary development [47]. Butyrate can also increase the blood flow during nutrient absorption and metabolism and can directly affect gene expression in the ruminal epithelium [4]. 

Rumen development can also be affected by the dietary nutrient level. Interestingly, lambs fed a high protein diet had a higher concentration of ammonia nitrogen (NH_3_–N) but a lower proportion of total VFA and propionate [48]. Moreover, study by Shen and coworkers (2004) [49] identified that a high energy diet lead to rumen papillae proliferation, which was associated with IGF-1 receptors and increased plasma IGF-1 levels in baby goats. However, excessive consumption of rapidly fermentable starter feed may predispose calves to rumen acidosis. Specifically, it can reduce ruminal pH, decrease rumen motility, and result in keratinization of papillae, causing a decreased in VFA absorption [42,50,51].

#### 3.1.3. Forage

Forage is less energy-intensive than starter feed. The low digestibility of forage in the rumen increases gut fill and decreases voluntary intake of concentrated feed by calves, which results in insufficient levels of VFAs required to stimulate rumen growth [52]. However, forage consumption is associated with positive effects of fiber on rumination and salivation in the GI tract [53,54]. The inclusion of forage in the diet increases rumen pH in both pre-weaning and post-weaning calves [55,56]. Importantly, intake of forage was negatively correlated with the severity of subacute ruminal acidosis (SARA), suggesting that a small quantity of consumed forage (0.080 kg/day) can alleviate rumen acidosis in calves [57]. The empty rumen weight was greater in calves supplemented with hay compared to calves fed a hay-free diet [54,56]. During weaning transition, feeding dietary forage in calves mitigates ruminal acidosis and induces changes in ruminal bacterial diversity and abundance [58]. Thus, two completely opposite opinions exist as to whether to feed forage to calves before weaning. To address this issue, several studies have been conducted to compare the effect of different initial time of forage provision on growth and rumen development in calves. Lin and colleagues (2017) [59] indicated that supplementation of oat hay to pre-weaned calves increased starter feed intake, ruminal pH, and reduced non-nutritive oral behaviors. Calves with hay supplementation initiated at two weeks of age showed the best productivity. Another study found that feeding forage to calves, either from 3 or 15 days of age, had no effect on growth rate, feed intake and rumen fermentation parameters compared to calves fed no forage, which also justified the supply of forage to young calves [60]. Inclusion of forage in the starter feed was positively linked with muscular development of the rumen [61,62] and morphological appearances of rumen epithelial cells, and caused decreased plaque formation [40,61]. Replacing 50% barley or corn with corn silage in the diet given to 10- or 90-day-old calves improved the thickness of the rumen wall, but had no significant effect on the papillae [63].

Different forage sources have different effects on stimulating chewing activity and saliva production [64]. Supplementation with NDF from alfalfa hay in the starter diet was shown to be more effective than beet pulp in increasing rumen pH and stimulating chewing activity [65]. A recent meta-analysis indicated that forage consumption can affect starter feed intake and performance in calves, which was modulated by forage level, sources, and physical forms of the starter [66]. 

#### 3.1.4. Physical Form

The physical form and particle size distribution of the diet exert significant influence on the anatomical and microbial development of the rumen [50,54]. For example, calves fed a ground diet had shorter papillae with a smaller surface area compared to calves fed the unground diet. Moreover, a decrease in cellulolytic bacteria and an increase in amylolytic bacteria were detected in calves fed the ground diet [50]. Consumption of finely ground diets can reduce ruminal pH [57] and lead to rumen parakeratosis [50,67]. Given these considerations, 75% of the particles in the starter feed should exceed 1190 μm in diameter [68]. Work by Lesmeister and Heinrichs (2004) [69] reported that calves fed texturized starter feed containing whole corn had higher ruminal pH compared to calves fed diet with dry-rolling corn, roasted-rolling corn, or steam-flaked corn. Increasing particle score of alfalfa hay from 1 mm to 3 mm can affect non-nutritive oral behaviours in calves fed finely ground starter feed [70]. However, research by Suarez-Mena and coworkers (2015, 2016) [8,71] suggested that increasing particle size of the starter diet by adding whole oat or straw of different lengths had no effect on rumen fermentation and calf development. Moreover, chopping of hay grass (~50% particles > 1.9 cm) decreased chewing time of calves [72], meanwhile, the richness and diversity of rectal microflora was reduced [73]. Provision of rations containing finely ground hay (2 mm) to calves may increase feed sorting and result in imbalanced intake of nutrients after weaning [74]. Increasing length of chopped hay from 2 mm to 3–4 cm reduced non-nutritive oral behaviors and improved nutrient digestibility [75]. The effect of the physical form and shape of the diet on calves is closely related to the inclusion rate, source, nutrient matrix and processing method of each ingredient. Importantly, the optimal calf diet specification designed specifically to promote rumen development has not been yet defined.

### 3.2. Feed Additives

#### 3.2.1. Probiotics

Probiotics are viable and beneficial microorganisms that help maintain GI microbial balance and promote rumen development. Feeding probiotics to calves around weaning age may facilitate the development of rumen bacterial communities and help calves with a transition from liquid feed to dry feed and forage [76,77]. Fermentation products of *Saccharomyces cerevisiae* have been shown to positively influence ruminal microbiota and improve ruminal morphology [78,79]. Specifically, effects of *Bacillus licheniformis*, *Saccharomyces cerevisiae* and their compounds can increase nitrogen utilization of the rumen microbial community and affect the fermentation pattern which was shown to be beneficial for growth of fattening lamb [80]. An oral dose of *Megasphaera elsdenii* NCIMB 41125 given to calves at 14 days of age increased ruminal butyrate, reticulorumen weight and papillae growth, suggesting an improvement in epithelial metabolism [81]. Supplementation of *Bacillus subtilis natto* in starter feed was shown to aid the development of rumen bacterial communities by increasing the growth of cellulolytic bacteria in calves after weaning [82].

However, feeding probiotics to calves has not always been shown to exert positive effects on the development of cellulolytic bacteria. For example, adding a mixture of *Lactobacillus plantarum* and *Bacillus subtilis* to MR and starter feed affected the denatured gradient gel electrophoresis (DGGE) fingerprint of the 16S ribosomal RNA genes, and reduced the number of *Ruminococcus albus* in calves [83]. In contrast, other studies reported that pH and enzymatic activities of rumen fluid were unaffected by three kinds of probiotic feeding in newborn calves [84]. Supplementation of *Candida tropicalis* in MR had no effect on the morphology of the forestomach and enzymatic activities of ruminal digesta [85]. Rumen and papillae measurements of Holstein bull calves were not affected by inclusion of *Aspergillus oryzae* fermentation extract in MR and starter feed [86]. Overall, the effects of probiotics on rumen development in calves are inconclusive, and frequently driven by differences in viable probiotic bacterial numbers, probiotics species, administration methods, and health status of animals. 

#### 3.2.2. Effects of VFAs

VFAs are the primary products of rumen fermentation and contribute to rumen epithelium development in calves. Previous studies suggested that infusion of sodium propionate or sodium butyrate greatly promotes the development of the rumen papillae in calves [6,42,87]. Supplementation of MR with sodium butyrate was associated with increased reticulorumen weight and increased length and width of papillae [37,88,89]. Another study showed that calves receiving a blend of short and medium chain fatty acids as monoglycerides (0.2%) in MR had less degenerative tissue accumulation and a higher number of cytoplasmic protrusions on the exposed horn surfaces [90]. 

Branched-chain VFAs (BCVFA), such as isobutyrate, isovalerate and 2-methylbutyrate, are naturally derived from the catabolism of branched-chain amino acids. Adequate levels of BCVFA are essential for the growth of some cellulolytic bacteria and digestion of structural carbohydrates in the rumen [91,92,93]. Supplementation of isobutyrate and isovalerate in milk and concentrate feed can accelerate the growth of calves by improving ruminal fermentation, rumen enzyme activities and growth of cellulolytic bacteria [94,95]. Administration of VFAs have been proved to be effective in promoting rumen development in calves, however, the optimal inclusion rate of VFAs and BCVFA in feed deserved further researches.

#### 3.2.3. Plant Extracts

There are many studies focused on evaluating the potential of plant extracts as alternatives to feed antibiotics and growth promoters in ruminant nutrition. Plant extracts have been shown to favorably affect rumen microbiota [96] and modulate ruminal fermentation in ruminants [97,98,99]. However, studies evaluating how plant extracts affect rumen development in young ruminants are limited. A recent study revealed that adding Aloe barbadensis to milk was beneficial in increasing total VFA concentration and bacterial count in cross-bred calves [100]. Supplementation of mulberry leaf flavonoids in MR increased α-amylase activity in ruminal digesta and protease activity in abomasal digesta in calves [85]. Supplementation of caraway and garlic in concentrated feed can improve rumen fermentation parameters by increasing total VFAs, increasing rumen pH and decreasing rumen ammonia in growing buffalo calves [101]. Thyme and cinnamon essential oils were shown to decrease the molar proportion of acetate and lower the ratio of acetate to propionate, as well as increase the level of propionate in Holstein calves consuming a high-concentrate diet. Finally, cinnamon essential oil was shown to increase rumen molar concentration of butyrate [102]. Plant extracts are among the most promising alternatives to antibiotics due to their extensive biological effects, and can be used in calf feed to prevent diarrhea. However, the efficacy of plant extracts is subject to a series of factors, including the composition of active components, addition levels, and physiological status of animals. The use of different types of plant extracts at various inclusion rates in the diet deserves further research. Moreover, effects of plant extracts on the colonization of microbial populations remains to be determined in calves.

### 3.3. Feeding Management

Weaning age can influence the development of rumen in pre-ruminants. For example, calves weaned at six weeks of age had longer and wider papillae compared to calves weaned at nine weeks of age [103]. In early-weaned calves, the ruminal pH, molar proportion of acetate and the ratio of acetate to propionate were lower, but the molar proportion of propionate and butyrate were greater [104]. The β-diversity of ruminal microbiota shifts rapidly in calves weaned at six weeks, while a more gradual shift is observed in calves weaned at eight weeks [105]. The colonization pattern substantially differs between newborn goats reared naturally with the dam and those reared artificially with MR. A higher bacterial diversity was observed in natural-fed goats [106]. Compared with suckling feeding, bottle feeding mode tended to increase the number of potential pathogens and delay the establishment of anaerobic microbes in the gut of lambs [18]. The total rumen bacterial population of lambs grazing at pasture with the nursing mother was lower compared to lambs weaned at 21 or 35 days of age, whereas methanogens and protozoa population were lower in early-weaned lambs compared to grazing lambs [107]. Kehoe and coworkers (2007) [108], however, reported that weaning age had no effect on rumen papillae length, width or rumen wall thickness. Different weaning methods (conventional weaning or concentrate-dependent weaning) result in similar rumen development [109]. The development of ruminal microbiome was not affected by the weaning strategy, and there was no effect of gradual or abrupt weaning [110]. The difference may mainly be associated with rumen development status. Due to the differences in feeding and management during the pre-weaning period, rumen development of calves may vary in different experiments. Calves with a well-developed rumen are able to utilize grains and forage efficiently. The effect of weaning age may only be detected in calves with undeveloped rumen. Additionally, pair-housed calves were shown to consume more solid feed at an earlier age compared to calves housed individually [111,112]. 

Intensive feeding of milk or MR may decrease starter feed intake, thereby delaying rumen development (Cowles, 2006) [113]. Hence, the amount of milk supplied to calves is normally restricted to promote starter feed intake and rumen development in conventional feeding practices [3,114]. However, calves fed limited amounts of milk had lower growth rates and abnormal behavior due to reduced nutrient intakes [55]. Schäff and colleagues (2018) [115] reported that compared to calves fed MR ad libitum, calves fed a restricted amount of MR had greater density of the rumen papillae in the atrium and ventral blind sac, but lower villus surface area and villus height/crypt depth ratio in the distal jejunum. Enhanced MR feeding increased the concentration of plasma IGF-1 and insulin [116,117], which may be beneficial for gastrointestinal growth in pre-weaning calves [16,28]. Furthermore, increasing nutrient intake from milk or MR resulted in enhanced milk yield in the first lactation [118]. Thus, intensive feeding practices have been widely adopted by producers; however, supporting feeding programs, such as a gradual weaning plan, need to be detailed to ensure optimum rumen development.

## 4. Conclusions

To summarize, it is beneficial for rumen development for calves to be fed high-quality liquid feed rich in biologically active substances. Minimization of the use of soy protein or appropriate acidification of MR may alleviate gastrointestinal epithelium lesions. Feeding readily fermentable carbohydrates to calves to increase VFA production can stimulate rumen development. A pellet or texturized starter feed is superior to a finely ground meal. Providing calves with high-graded forage, such as alfalfa hay, can reduce the occurrence of rumen acidosis and papillae keratinization. Moreover, additives can be used in calf feed due to their potential advantages in rumen manipulation, however, the types and the optimal inclusion rate deserve further study. More importantly, there is no fixed pattern of calf feed. The diet compositions and nutrient specifications should be matched with the feeding program and management to better promote rumen development.

The rumen is a unique part of the GI tract in ruminants. As the rumen develops and becomes colonized by microorganisms, a calf physiologically transitions from a pseudo-monogastric to a functioning ruminant. The development of rumen in calves can directly affect feed intake, nutrient digestibility and eventual growth of calves. Any changes in the early feeding regime and nutrition can influence rumen development, and thus, lead to long-lasting effects on subsequent growth, health, and milk production performance. Study by Moallem and colleagues (2010) [29] reported higher milk yields during the first lactation in heifers fed whole milk compared to heifers fed MR. Moreover, the same study suggested that MR did not impart any milk-borne effects in calves [29]. Increasing the nutrient intake from milk or MR prior to weaning resulted in an increased milk yield during the first lactation [118,119,120]. This phenomenon may be associated with epigenetic effects of early nutrition [118].

Additionally, an early feeding regime and nutrition can influence rumen development and rumen microbial composition, ultimately exerting an effect on the lifetime milk yield in cattle. Studies indicated that diets can modify the establishment of the bacterial community in lambs during weaning, which can persist for four to five months [121,122]. The postnatal period is frequently referred to as the most sensitive window for rumen manipulation [123,124,125], although studies evaluating ruminal imprinting are still limited. The majority of published studies focus on rumen organ development, rumen fermentation parameters, morphology, and changes in the population of cellulolytic bacteria. With the development of microbial 16S rDNA gene sequencing and metagenome analysis, additional studies will likely reveal the interactions between host GI tract development and establishment of rumen bacteria.

## Figures and Tables

**Table 1:** The development of the forestomach.

Items ^1^	0 w	8 w	12–16 w
Reticulorumen %	38	61.23	67
Omasum %	13	13.4	18
Abomasum %	49	25.37	15
Total	100	100	100

^1^ Each stomach compartment is expressed as a percentage of the total weight of the forestomach.
